# Carbon Dots Intercalated MXene for Flexible Organic Hydrogel Absorbers with Synergistically Enhanced Dielectric Loss

**DOI:** 10.1007/s40820-026-02135-6

**Published:** 2026-03-25

**Authors:** Bokai Lu, Guangkai Jin, Yuhong Cui, Tianyi Zhang, Shujuan Liu, Qian Ye, Xuqing Liu, Feng Zhou

**Affiliations:** 1 State Key Laboratory of Solidification Processing, Center of Advanced Lubrication and Seal Materials, School of Materials Science and Engineering, Northwestern Polytechnical University, Xi’an, 710072 People’s Republic of China; 2 State Key Laboratory of Solid Lubrication, Lanzhou Institute of Chemical Physics, Chinese Academy of Sciences, Lanzhou, 730000 People’s Republic of China

**Keywords:** Carbon dots, MXene, Organic hydrogel, Microwave absorber

## Abstract

With the rapid development of flexible electronics and wearable devices, there is an increasing demand for absorbing materials that exhibit high electromagnetic wave absorption efficiency and mechanical flexibility. In this work, we present a novel and effective strategy for fabricating flexible microwave absorbers via synergistically integrating a polymer framework with conductive fillers. Carbon dots (CDs) functionalized with catechol groups were first synthesized via a bottom-up approach using directed ultrasonication, employing tannic acid dispersed in acetone as the precursor. Owing to the abundant surface functional groups, the as-prepared CDs were uniformly anchored onto MXene nanosheets through chemical bonding, forming a well-dispersed MXene/CDs composite. Subsequently, this hybrid filler was incorporated into a polymer network composed of polyvinyl alcohol (PVA) and acrylamide, with water and glycerol as co-solvents, to fabricate a flexible MXene/CDs organic hydrogel. Notably, glycerol plays an important role in adjusting the polarity of the gel system, thereby effectively optimizing impedance matching. Meanwhile, the abundant heterogeneous interfaces between MXene and CDs significantly enhanced interfacial polarization, and the synergistic coupling of optimized impedance matching with strong dielectric loss endowed the MXene/CDs organic hydrogel with outstanding electromagnetic wave absorption performance. The absorber achieves a minimum reflection loss (RL_min_) of − 47.9 dB at 9.46 GHz with a matching thickness of 3.1 mm, along with an effective absorption bandwidth of 3.5 GHz. Furthermore, the synergistic reinforcement of dual-crosslinked polymer chains and the MXene/CDs filler endowed the hydrogel with excellent mechanical robustness, making it suitable for flexible and wearable absorption applications.

**Supplementary Information:**

The online version contains supplementary material available at 10.1007/s40820-026-02135-6.

## Highlights

- Catechol-functionalized carbon dots chemically anchor onto MXene nanosheets, enhancing interfacial polarization and enabling impedance matching optimization through glycerol-regulated gel polarity.
- A flexible organic hydrogel absorber was constructed via synergistic integration of a dual-crosslinked polymer network and conductive MXene/carbon dots (CDs) hybrid fillers.
- The MXene/CDs hydrogel exhibits outstanding performance (RL_min_= −47.9 dB, 3.5 GHz bandwidth at 3.1 mm) together with improved mechanical robustness.

**Supplementary Information:**

The online version contains supplementary material available at 10.1007/s40820-026-02135-6.

## Graphical abstract

**Figure:**
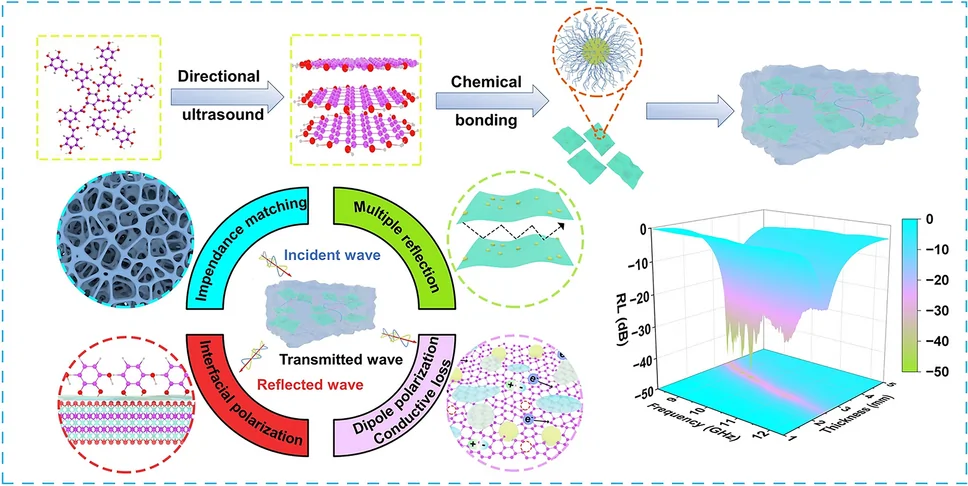

## Introduction

The rapid development of modern wireless communication technologies and the widespread use of electronic devices have made electromagnetic radiation and interference increasingly serious concerns. In military applications, there is a growing demand for effective countermeasures and stealth capabilities in complex electromagnetic environments. Meanwhile, the proliferation of wearable electronics in civilian applications imposes higher requirements for material flexibility and functional integration. Microwave-absorbing materials, a class of functional materials capable of effectively attenuating electromagnetic wave reflection, dissipate incident energy through dielectric and/or magnetic losses, converting it into thermal or other forms of energy [1–3]. Although traditional electromagnetic wave absorbers, such as carbon material [4, 5], metal material [6, 7] and conductive polymer with conjugated structures [8], can exhibit strong wave absorption performance, their practical use remains limited by rigid forms and insufficient multifunctionality. Consequently, the development of novel materials that integrate effective microwave absorption with good mechanical flexibility has become a research focus in the field of functional materials.

In recent years, gel-based flexible wave-absorbing materials have attracted increasing attention. The porous structure of hydrogel is beneficial for impedance matching [9], while water molecules can enhance dipolar polarization and thereby improve dielectric loss capability to some extent. In addition, their excellent flexibility, mechanical properties, and high structural tunability endow hydrogels with broader potential for multifunctional application [10, 11]. However, hydrogels using pure water as the sole solvent suffer from poor environmental tolerance. Water evaporation at elevated temperatures and freezing at low temperatures can lead to mechanical degradation, while the high polarity of water may induce impedance mismatch. To overcome these limitations, organic solvents like glycerol or dimethyl sulfoxide (DMSO) have been introduced to partially or completely replace water, yielding organic hydrogel with improved dielectric properties [12]. Nevertheless, a single gel network typically provides limited electromagnetic energy dissipation pathways, resulting in suboptimal absorption performance. Therefore, incorporating conductive fillers to construct heterogeneous interfaces is an effective strategy to regulate interfacial polarization, electronic band structure, and charge transport behavior, thereby enhancing electromagnetic energy dissipation.

Two-dimensional (2D) materials, such as MoS_2_, MXene, and grapheme, have shown great potential for electromagnetic wave absorption owing to their low density, high specific surface area, and favorable mechanical behaviors. Among them, MXene, as a 2D transition metal carbonitride, has high intrinsic electrical conductivity, extremely hydrophilicity, abundant surface groups (–OH, –F, =O), and a suitable density of crystal defects, which facilitate surface functionalization and composite construction for electromagnetic wave absorption [13–15]. For instance, Zhang et al. fabricated porous MXene nanoribbons via alkali intercalation, achieving a minimum reflection loss (RL_min_) of −38.14 dB, demonstrating notable microwave absorption performance [16]. Carbon dots (CDs), an emerging class of zero-dimensional carbon nanomaterial with sizes below 10 nm, consist of a carbon core surrounded by surface functional groups. Beyond their unique fluorescence properties, CDs feature excellent biocompatibility and versatile surface chemistry [17–19]. When integrated with MXene nanosheets, CDs can expand the MXene interlayer spacing and effectively inhibit its self-restacking. In addition, the ultrahigh specific surface area and abundant functional interfaces of CDs promote interfacial polarization within the composite filler, thereby improving electromagnetic wave attenuation. For example, Zhou et al. constructed a three-dimensional (3D) aerogel with abundant heterogeneous interfaces by integrating graphene quantum dots (GQDs) with MXene, when embedded in paraffin, the resulting composite exhibited an RL_min_ of −53.21 dB [20].

Catechol groups rich in polar hydroxyl groups can increase the surface defects and polarization capability of the CDs, thereby improving their dielectric response. Compared with gallic acid and catechol, tannic acid (TA), featuring a glucose core linked to multiple gallic acid units, offers a higher catechol density and a more defect-rich framework, enabling simultaneous CDs nucleation, defect engineering, and surface functionalization in a single step. Catechol-functionalized CDs derived from TA have previously been synthesized using hydrothermal [21] and laser-irradiation method [22]. In this work, CDs rich in catecholic groups were prepared via a bottom-up strategy using directed ultrasonication of TA as the precursor. The resulting CDs were anchored onto MXene nanosheets through chemical bonding between the catechol groups on the CDs and the surface Ti–OH groups of MXene, yielding MXene/CDs composites. The as-prepared MXene/CDs were then incorporated into an organic hydrogel system composed of poly(vinyl alcohol) (PVA) and acrylamide (AAm), with water and glycerol as co-solvents, to fabricate a flexible MXene/CDs hydrogel microwave absorber. As a result, the MXene/CDs gel exhibited excellent impedance matching and outstanding electromagnetic wave absorption performance, achieving RL_min_ of − 47.9 dB at 3.1 mm thickness with an absorption bandwidth of 3.5 GHz. Furthermore, the MXene/CDs gel displayed improved mechanical performance, with a fracture strength of 0.196 MPa and a fracture elongation of 493.8%.

## Experimental Section

### Materials

LiF, sodium tetraborate, *N*,*N*′-methylenebisacrylamide (MBAA), Tannic acid (TA), HCl, and glycerol were obtained from Energy Chemical Company. MAX (Ti_3_AlC_2_) was purchased from Jinzhou Haixin Metal Materials Co., Ltd. Poly(vinyl alcohol) (PVA), Ammonium persulfate (APS), and acrylamide (AAm) were purchased from Aladdin Biochemical Technology Co., Ltd.

### Preparation of MXene Nanosheets

Single- or few-layer MXene nanosheets were prepared according to previously reported methods [23]. Briefly, 6 g of LiF was dissolved to 120 mL of 9 M HCl under continuous stirring until complete dissolution. Subsequently, 6 g of MAX phase powder was gradually added to the solution, and the etching reaction was maintained at 35 °C for 24 h. After etching, the resulting suspension was repeatedly washed with deionized water until the supernatant became viscous and reached neutral pH. The obtained sediment was then ultrasonicated for 45 min to exfoliate multilayered MXene into single- or few-layer MXene nanosheets. Finally, the dispersion was centrifuged at 3500 rpm for 2 min, and the supernatant containing the MXene nanosheets was collected for further use.

### Preparation of MXene/CDs Composites

Carbon dots (CDs) were first prepared by dispersing 50 mg of TA in 10 mL of acetone using ultrasonication. The mixture was then subjected to directed ultrasonication in an ice bath at 0 °C under pulse mode (2:1 duty cycle, 40% amplitude). After sonication, the suspension was centrifuged at 10,000 rpm for 10 min, and the supernatant containing the CDs was collected.

For the preparation of MXene/CDs composites, 15 mL of the as-prepared MXene solution (6 mg mL^−1^) was mixed with 3.6 mL of the CDs solution (5 mg mL^−1^) and magnetically stirred for 18 h. During this process, the catechol groups on the CDs formed bidentate coordination bonds with Ti atoms on the MXene surface, enabling the uniform anchoring of CDs onto the MXene nanosheets. The resulting suspension was then vacuum-filtered and freeze-dried to obtain the MXene/CDs composite.

### Preparation of MXene/CDs Organic Hydrogel

The MXene/CDs organic hydrogel was prepared via free radical polymerization. Briefly, 0.7 g of AAm and 0.2 g of PVA were dispersed in 5 mL of a glycerol/water mixed solvent (1:1, v/v). Subsequently, 10 mg of the MXene/CDs composite was added and uniformly dispersed. Then, 90 μL of MBAA aqueous solution (10 wt%), 90 μL of APS aqueous solution (10 wt%), and 500 μL of sodium tetraborate aqueous solution (4 wt%) were successively added. After thorough mixing, the precursor solution was poured into a polytetrafluoroethylene (PTFE) mold and polymerized at 85 °C to form MXene/CDs gel. For comparison, blank gel was synthesized under identical conditions without the addition of MXene/CDs, while a MXene gel was prepared by adding an equivalent mass of pure MXene nanosheets.

## Results and Discussion

### Preparation of MXene/CDs and Gel

**Fig. 1:**
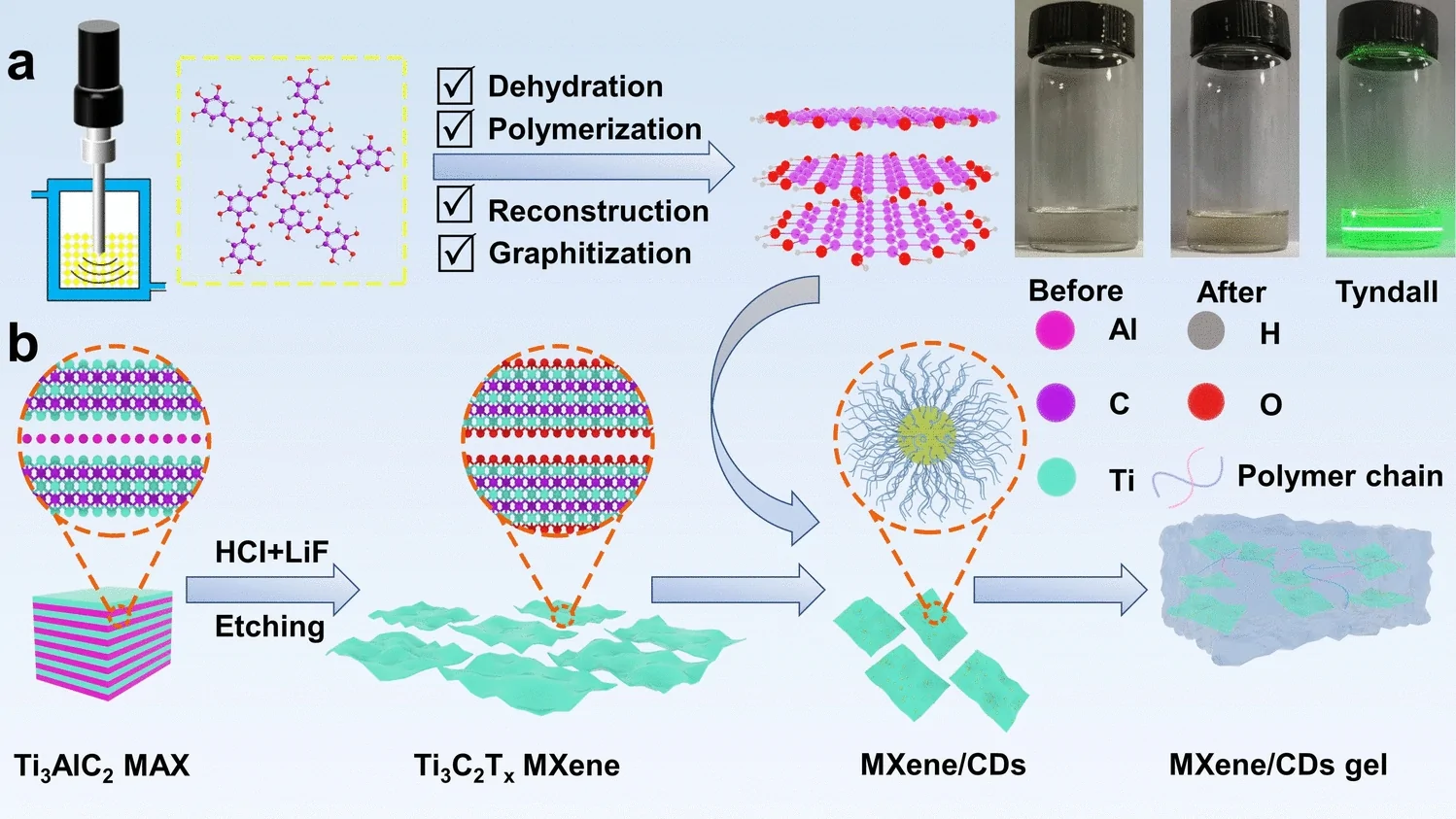
Schematic preparation of **a** CDs and **b** MXene/CDs gel

The preparation of CDs via directed ultrasonication is illustrated in Fig. 1a. High-power ultrasound generates substantial instantaneous localized heat, creating an extreme non-equilibrium environment. Under these conditions, TA molecules dispersed in acetone undergo dehydration, polymerization, reorganization, and graphitization, ultimately forming CDs with a carbon core surrounded by surface catechol groups. Multilayered MXene nanosheets were obtained from the MAX phase precursor through a selective etching strategy using concentrated HCl and LiF. Subsequently, ultrasound treatment exfoliated the multilayered MXene into single- or few-layer nanosheets decorated with abundant active groups such as –OH, –F, and = O [24]. The CDs were then anchored onto MXene nanosheets via bidentate coordination interactions between the catechol groups of CDs and Ti–OH bonds on the MXene surface, resulting in the formation of MXene/CDs composite. As shown in Fig. 1b, the MXene/CDs composite was incorporated into a mixed solvent system of water and glycerol containing PVA and AAm monomers, followed by the sequential addition of the chemical crosslinker MBAA, initiator APS, and borax. Free radical polymerization at elevated temperatures yielded the MXene/CDs organic hydrogel with a 3D network structure.

### Characterization of MXene/CDs

**Fig. 2:**
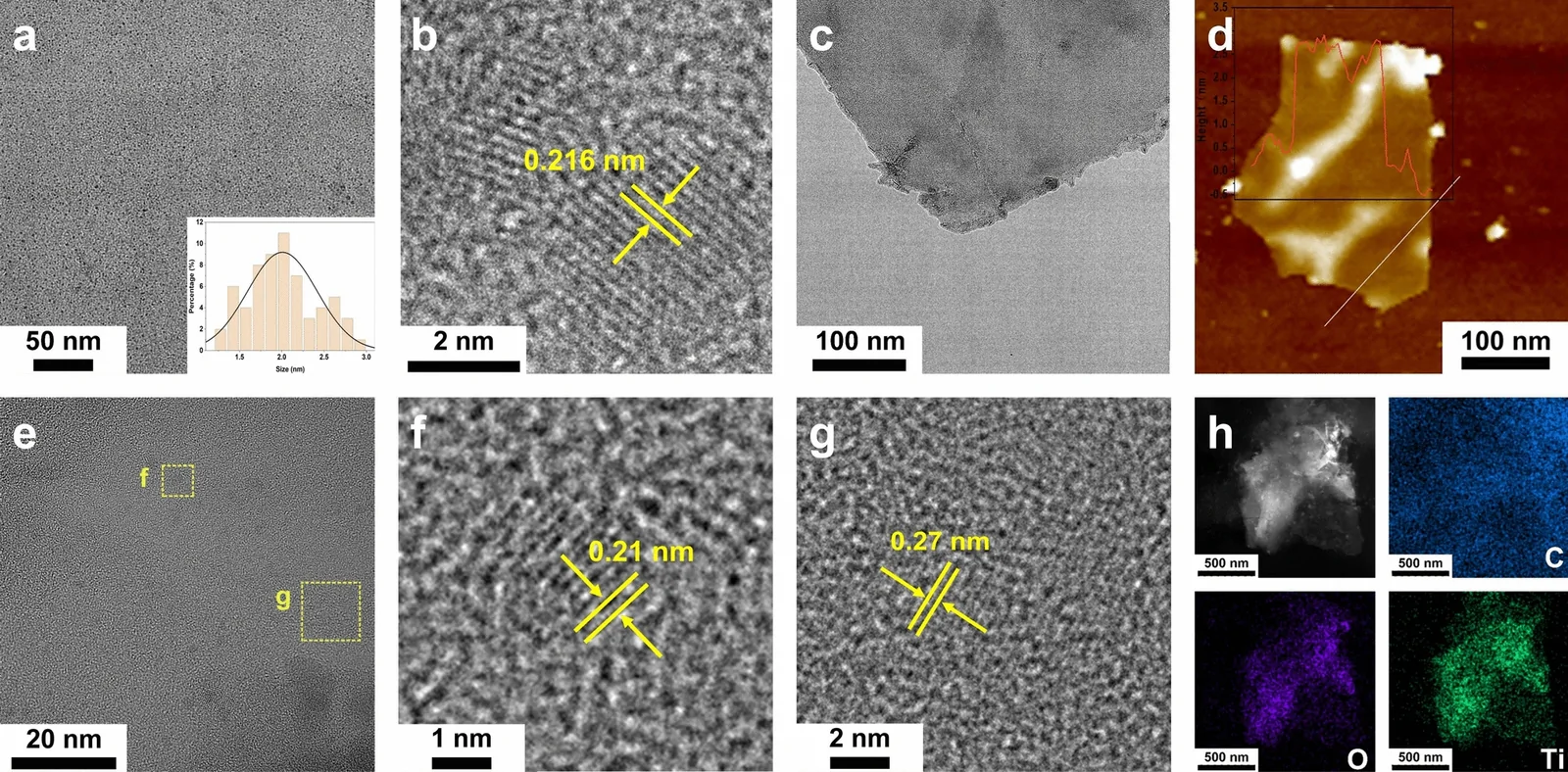
**a** TEM, **b** HR-TEM images of CDs. **c** TEM, **d** AFM images of MXene. **e** HR-TEM image of MXene/CDs. **f**, **g** Corresponding dotted regions in **e**, **h** HAADF image and element mappings of MXene/CDs

TEM image shows that the CDs prepared via directed ultrasonication exhibit a spherical morphology with a mean diameter of approximately 2 nm (Fig. 2a). The High-resolution TEM (HR-TEM) image in Fig. 2b reveals well-defined lattice fringes with a spacing of 0.216 nm, which can be assigned to the (100) plane of graphitic carbon [25]. XPS survey spectra indicate that no new elements were introduced during the transformation of the TA precursor into CDs; however, the oxygen content increased significantly. Deconvolution of the C 1*s* spectra shows a higher proportion of C–OH bonds in the CDs compared to the TA precursor, confirming the successful formation of CDs enriched with oxygen-containing functional groups via directed ultrasonication (Fig. S1). UV–Vis spectroscopy further supports the successful synthesis: the TA precursor displays negligible light absorption, whereas the CDs exhibit a distinct absorption peak at 375 nm (Fig. S2).

The morphology of the single- or few-layer MXene was characterized by TEM and atomic force microscopy (AFM). The MXene nanosheets display an ultrathin, sheet-like structure with well-defined edges and an average thickness of about 3 nm (Fig. 2c, d). HR-TEM of the MXene/CDs composite reveal lattice spacings of 0.21 and 0.27 nm, corresponding to the (100) plane of graphitic carbon and MXene (Fig. 2e–g) [26]. Elemental mapping analysis (Fig. 2h) shows a homogeneous distribution of Ti and O elements across the MXene nanosheets, indicating the successful anchoring of CDs on the MXene surface.

**Fig. 3:**
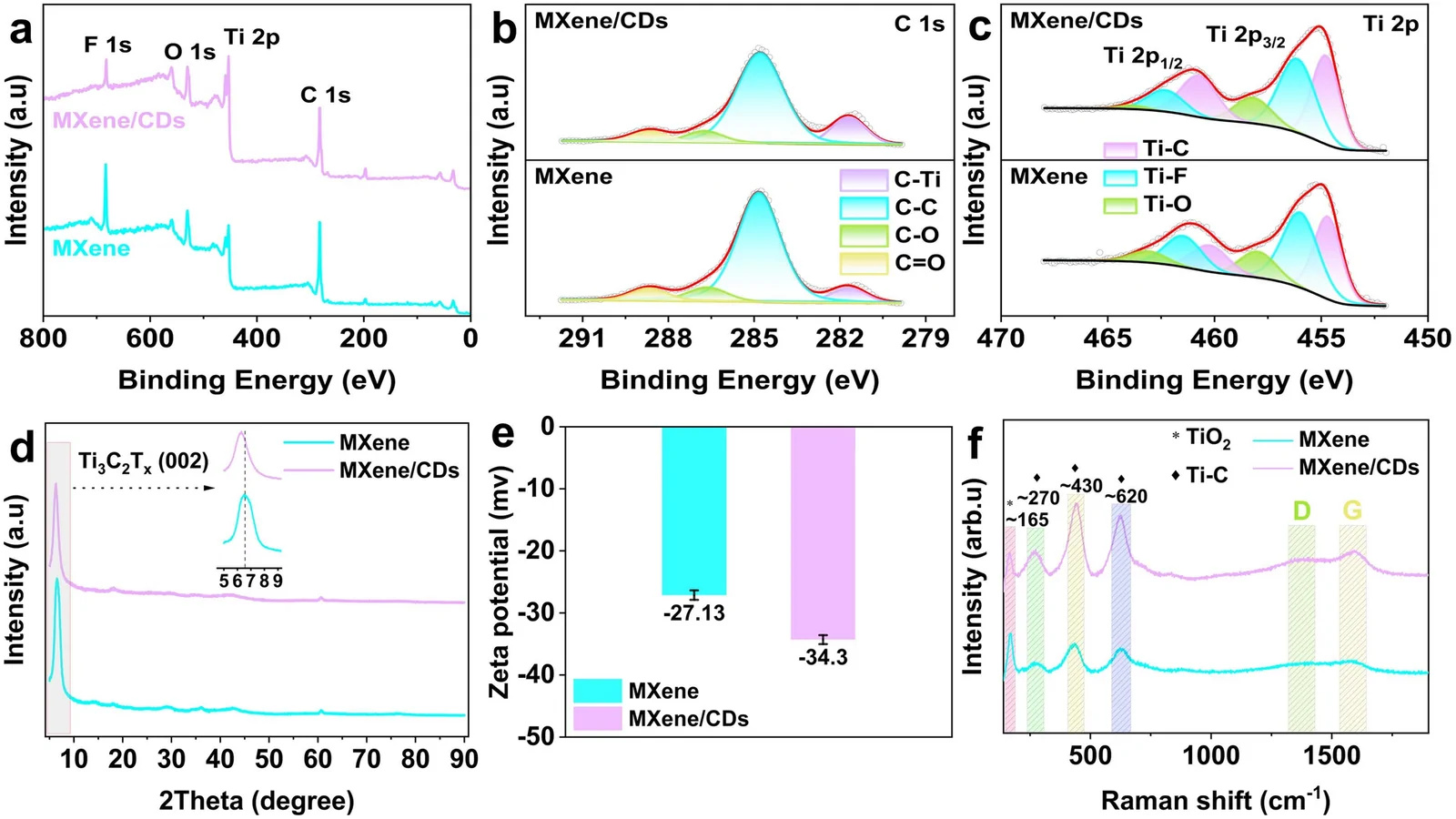
**a** XPS full spectra, high-resolution spectra of **b** C 1*s* and **c** Ti 2*p* of MXene and MXene/CDs. **d** XRD pattern, **e** Zeta potential, **f** Raman spectra of MXene and MXene/CDs

To further confirm the successful loading of CDs onto MXene nanosheets, a series of structural and surface characterizations were performed. SEM images reveal that the pristine MAX phase exhibits a typical layered bulk morphology, whereas selective etching of the Al layers produces 2D MXene nanosheets. Importantly, the introduction of CDs does not disrupt the structural integrity of the MXene nanosheets (Fig. S3). XPS survey spectra show that no new elements were introduced after CDs loading, which is consistent with theoretical expectations (Fig. 3a). High-resolution C 1*s* spectra exhibit four characteristic peaks at approximately 281.6, 284.8, 286.8, and 288.6 eV, corresponding to C–Ti, C–C, C–O, and C=O bonds [27]. The Ti 2*p* spectrum can be deconvoluted into six characteristic peaks assigned to Ti–C 2*p*_3/2_ (454.8 eV), Ti–F 2*p*_3/2_ (456.2 eV), Ti–O 2*p*_3/2_ (458.2 eV), Ti-C 2*p*_1/2_ (460.8 eV), Ti–F 2*p*_1/2_ (462.3 eV), and Ti–O 2*p*_1/2_ (463.8 eV), respectively. Notably, all six peaks in the MXene/CDs composite exhibit slight positive shifts relative to pristine MXene (Fig. 3b, c), reflecting the influence of chemical bonding between MXene and CDs on the binding energy of the Ti atomic orbitals [28]. The O 1*s* spectrum was deconvoluted into three peaks at 529.5, 531.8, and 533.3 eV, ascribed to TiO_2_, Ti–O, and –OH species, respectively. Compared with pristine MXene, the relative proportion of TiO_2_ after CDs loading, indicating mild surface oxidative induced by the incorporation of CDs (Fig. S4) [29].

The XRD patterns in Fig. 3d show a strong characteristic peak at approximately 6.58°, corresponding to the (002) crystal plane of MXene, confirming the successful preparation of high-purity few-layer MXene nanosheets. After CDs loading, the (002) peak shifted slightly toward lower angle, indicating that the introduction of CDs not only preserved the layered structure of MXene but also expanded its interlayer spacing [30]. The abundance of surface groups (–OH, –F, and =O) on MXene result in a negative zeta potential of − 27.13 mV (Fig. 3e), which became more negative upon CDs loading due to the presence of catechol groups on CDs [31]. Raman spectroscopy provides additional insights into structural changes. The D and the G band both intensify after CDs loading, indicating an increase in structural defects and graphitic order. In addition, characteristic peaks located at near 165, 270, 430, and 620 cm^−1^ are observed, which are associated with TiO_2_ and Ti–C bonds and reflect the surface functional groups information of MXene (Fig. 3f) [16, 32, 33]. Fourier transform infrared (FTIR) spectra were employed to examine the surface chemical environment of MXene and MXene/CDs composites. The signal near 550 cm^−1^ originates from the Ti–C bond, while the absorption band at 1630 cm^−1^ is attributed to the Ti–O bond. The broad band observed between from 3100 to 3650 cm^−1^ indicates the presence of hydrogen bonding interactions within MXene. After CDs loading, a new absorption peak appeared at 2950 cm^−1^ in MXene/CDs composite, ascribing to the C–H stretching vibration from the CDs (Fig. S5) [34]. Together, these findings further confirm the successful anchoring of CDs onto MXene nanosheets.

### Characterization of MXene/CDs Gel

**Fig. 4:**
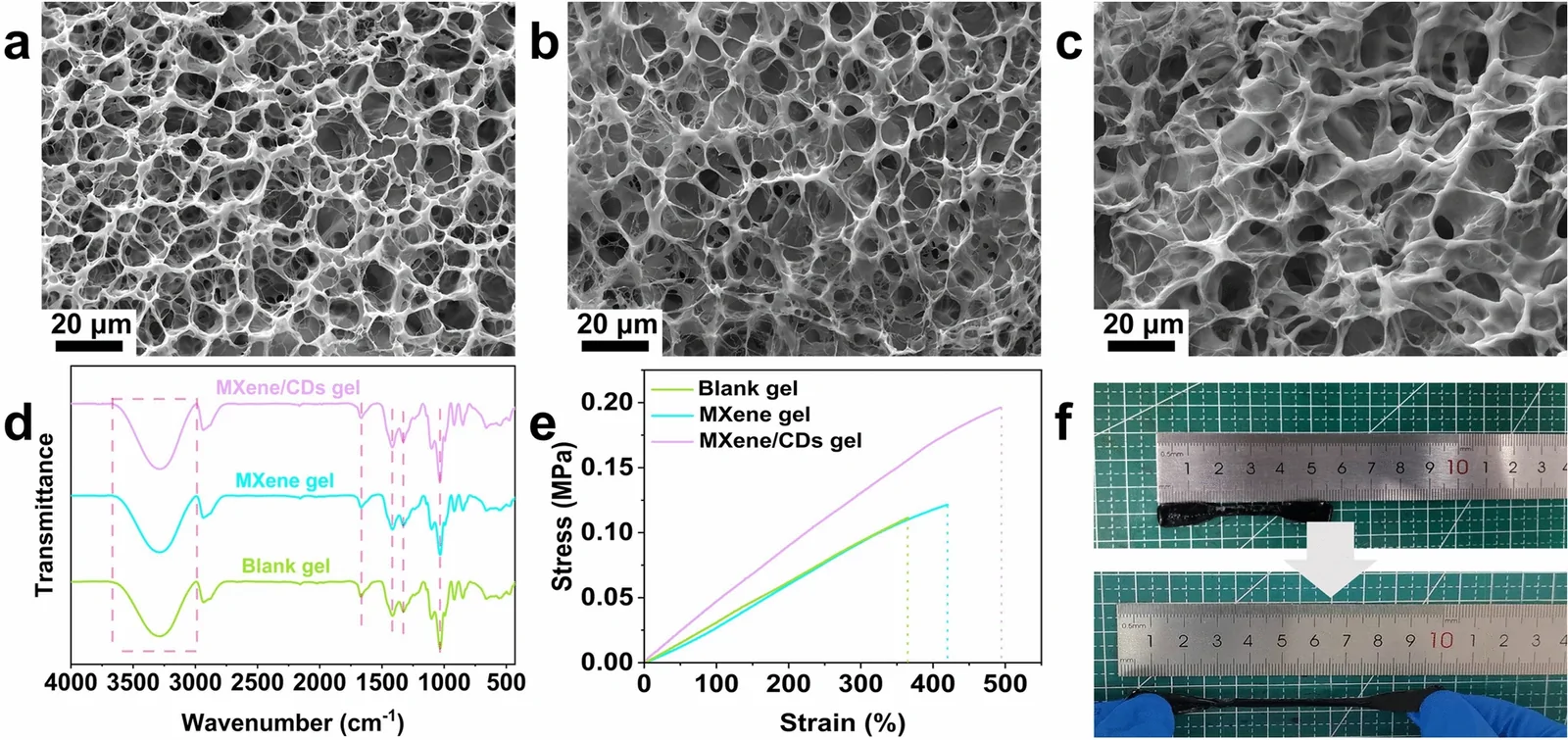
SEM images of the dry gel morphology of **a** blank gel, **b** MXene gel and **c** MXene/CDs gel. **d** FTIR spectra and **e** stress–strain curves of blank gel, MXene gel and MXene/CDs gel. **f** Stretch image of MXene/CDs gel

Figure 4a–c presents the SEM images of the freeze-dried hydrogels, revealing a well-defined 3D porous network structure formed by interconnected polymer chains. In contrast, in their natural hydrated state (Fig. S6), the gels exhibit a wrinkled morphology without discernible pores, which can be attributed to the strong hydrogen bonding interactions between the hydrophilic polymer network and the water/glycerol molecules [35]. Notably, after the incorporation of MXene and MXene/CDs, no significant changes in the surface morphology were observed, indicating good compatibility between the MXene/CDs fillers and the hybrid solvent-polymer matrix. To further investigate the internal interactions within the hydrogels, FTIR spectroscopy was performed. The characteristic peak at 1037 cm^−1^ is attributed to the vibrational absorption of glycerol, while those at 1330 and 1420 cm^−1^ owed to the − OH and –CH_2_ groups of glycerol, respectively. The peak near 1670 cm^−1^ arises from the asymmetric stretching vibration of C=O in the polyacrylamide (PAAM) polymer chains. Additionally, the broad absorption peak spanning 3000–3500 cm^−1^ corresponds to hydrogen-bonded -OH and -NH₂ groups, indicating strong non-covalent interactions between polymer chains and solvent molecules in the hydrogel network (Fig. 4d) [36]. The mechanical properties of the hydrogels were evaluated by tensile testing to assess their potential for practical applications. As displayed in Fig. 4e, the blank gel exhibits a fracture strength of 0.112 MPa and a fracture elongation of 365.2%. Upon incorporation of MXene, these values increase to 0.122 MPa and 420%, respectively. Notably, the MXene/CDs organic hydrogel demonstrates a substantial enhancement in mechanical performance, achieving a fracture strength of 0.196 MPa and a fracture elongation of 493.8%. This improvement can be attributed to the incorporation of MXene and CDs, which introduce additional physical crosslinking sites and thereby increase the overall crosslinking density of the gel. Beyond their inherent mechanical reinforcement, these nanomaterials also provide supplementary pathways for energy dissipation during deformation [21]. Meanwhile, the abundant hydroxyl groups on their surface facilitate the formation of strong covalent and non-covalent interactions within the polymer network, further reinforcing the gel structure [37]. The macroscopic appearance of the MXene/CDs gel before and after stretching is shown in Fig. 4f, where no visible surface cracks were observed upon deformation.

### Microwave-Absorbing Ability of MXene/CDs Gel

**Fig. 5:**
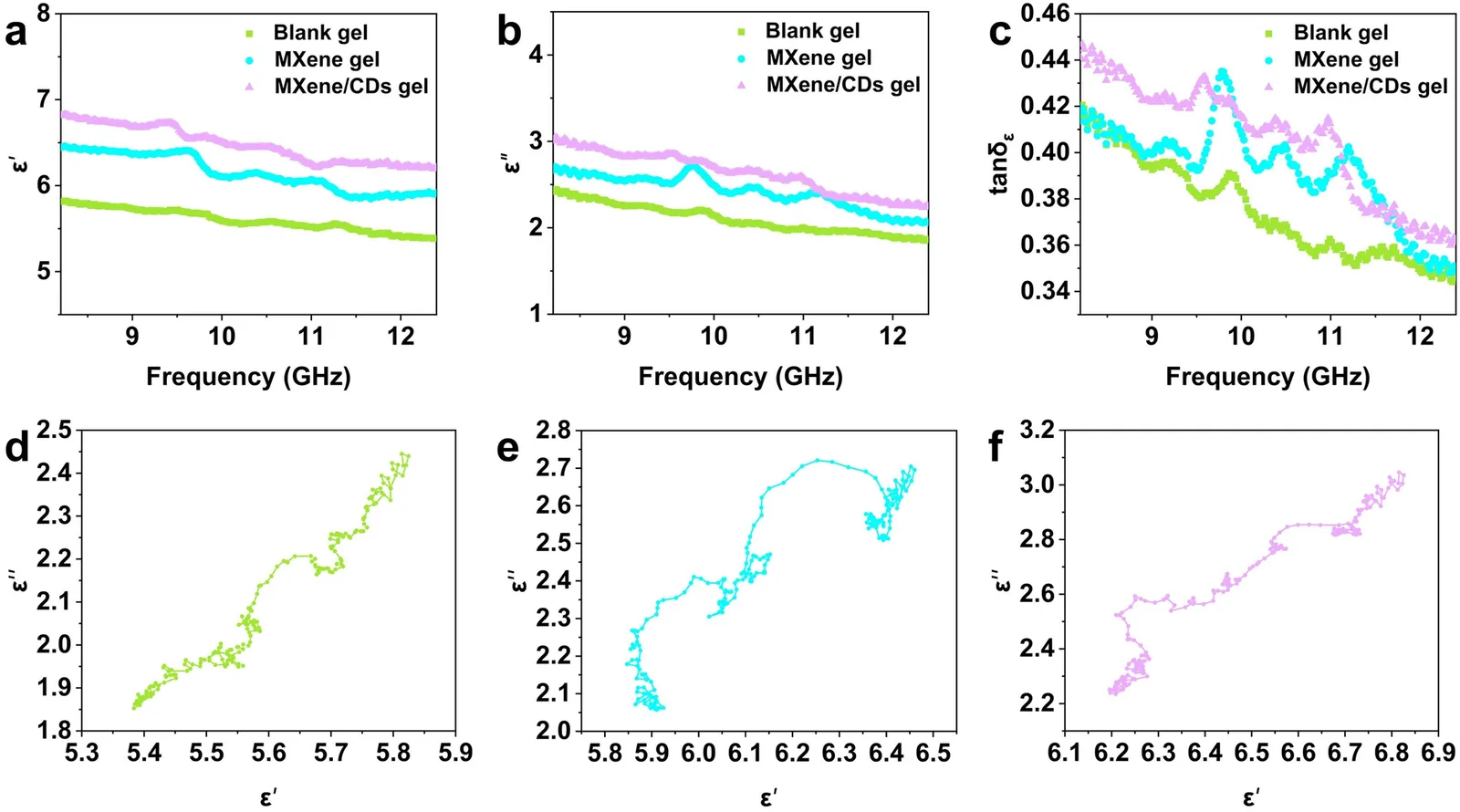
**a** *ε*′, **b** *ε″* and **c** tan*δ*_*ε*_ of blank gel, MXene gel and MXene/CDs gel varies with frequency. The *ε″*–*ε*′ curves of **d** blank gel, **e** MXene gel and **f** MXene/CDs gel

Electromagnetic wave-absorbing materials dissipate incident waves primarily by converting electromagnetic energy into thermal or other forms of energy through dielectric and magnetic losses. The absorption performance of such material is largely governed by their complex permittivity and complex permeability. The complex permittivity consists of a real part (*ε*′) and an imaginary part (*ε″*), where *ε*′ reflects the material's electrical energy storage capability under an external electric field, while *ε″* represents its ability to dissipate electric energy, which is closely related to electrical conductivity [38]. The dielectric loss tangent (tan *δ*_*ε*_ = *ε″*/*ε*′) is commonly used as an intuitive indicator of the material's dielectric loss capability [39]. Likewise, the complex permeability comprises a real part (*μ*′) and an imaginary part (*μ″*), corresponding to the storage and dissipation of magnetic energy, respectively, with the magnetic loss tangent (tan *δ*_*μ*_ = *μ″*/*μ*′) serving as a measure of magnetic loss [40]. As shown in Fig. 5a, the *ε*′ values of the blank gel, MXene gel, and MXene/CDs gel gradually decrease with increasing frequency, exhibiting a typical frequency‑dispersion behavior. This phenomenon arises from the delayed polarization relaxation under high‑frequency electromagnetic fields [41]. The incorporation of MXene into the blank gel results in a significant increase in both *ε*′ and *ε″*, indicating enhanced polarization capability and improved energy storage and dissipation. The increase in *ε*′ is primarily attributed to the strong interfacial polarization induced by the MXene incorporation. Upon further loading CDs onto MXene, both *ε*′ and *ε″* increase again (Fig. 5b), indicating that the introduction of MXene and CDs synergistically enhance interfacial polarization and charge storage capacity within the gel matrix (Fig. 5b). The tan *δ*_*ε*_ curves of all gels are shown in Fig. 5c. The MXene/CDs gel exhibits the highest tan *δ*_*ε*_, indicating the strongest dielectric loss capability. Consistently, the attenuation coefficient α of the three gels (Fig. S6) further follows the same trend, with the MXene/CDs gel displaying the largest α value, indicating its superior electromagnetic waves attenuation performance [42]. Because all gels are composed of nonmagnetic components, their *μ*′ and *μ″* values remain close to 1 and 0, respectively (Fig. S7), confirming that electromagnetic loss is dominated by dielectric loss. As presented in Fig. S9, the electrical conductivities of the blank gel, MXene gel, and MXene/CDs gel are 1.8, 2.95, and 2.27 × 10^–4^ S m^−1^, respectively. The incorporation of MXene nanosheets significantly increases the conductivity of the blank gel, indicating the formation of an effective conductive network. The slight reduction in conductivity for the MXene/CDs gel is attributed to the intercalation of CDs between MXene layers, which suppresses MXene self-restacking. This structural regulation not only reduces surface reflection, but also contributes to improved impedance matching.

Dielectric loss generally arises from two primary mechanisms: conductive loss and polarization loss, the latter of which includes electronic, ionic, interfacial, and dipole polarization processes [43]. In the microwave frequency range, electronic and ionic polarizations occur at much higher frequencies and are therefore not readily activated. Therefore, the superior dielectric loss performance of the MXene/CDs gel primarily arises from conductive loss, interfacial polarization, and dipole polarization. Among these, conductive loss is mainly governed by the electrical conductivity of the material [44]. The interfacial polarization occurs at the interfaces between components with different conductivities or permittivities, where charge carriers accumulate to form macroscopic dipoles. Under an external electric field, these dipoles undergo relaxation processes that dissipate electromagnetic energy as heat. Dipole polarization stems from the reorientation of permanent dipole moments in polar molecules or functional groups under high-frequency electric field. The relaxation of these dipoles similarly converts electromagnetic energy into thermal energy. In the MXene/CDs gel system, conductive loss is primarily attributed to the high intrinsic electrical conductivity of MXene, while the *sp*^2^-hybridized graphitic structure of CDs provides additional conductive pathways. The abundant heterogeneous interfaces between the MXene and CDs, combined with the high specific surface area of CDs, significantly enhance interfacial polarization. Meanwhile, dipole polarization arises from polar solvent molecules (water and glycerol) as well as surface functional groups on MXene and CDs. Furthermore, crystal defects within the conductive network disrupt the charge balance and create localized charge separation, generating additional dipoles. Under microwave irradiation, the directional reorientation of these dipoles during polarization relaxation results in effective dissipation of electromagnetic energy as heat. The *ε*″–*ε*′ relationships of the as-prepared gels are depicted in Fig. 5d–f. The presence of multiple semicircular arcs, known as Cole–Cole semicircles, indicates multiple Debye relaxation processes within the materials [45]. In addition, regions where *ε″* varies linearly with *ε*′ suggest the formation of a continuous and effective conductive network.

**Fig. 6:**
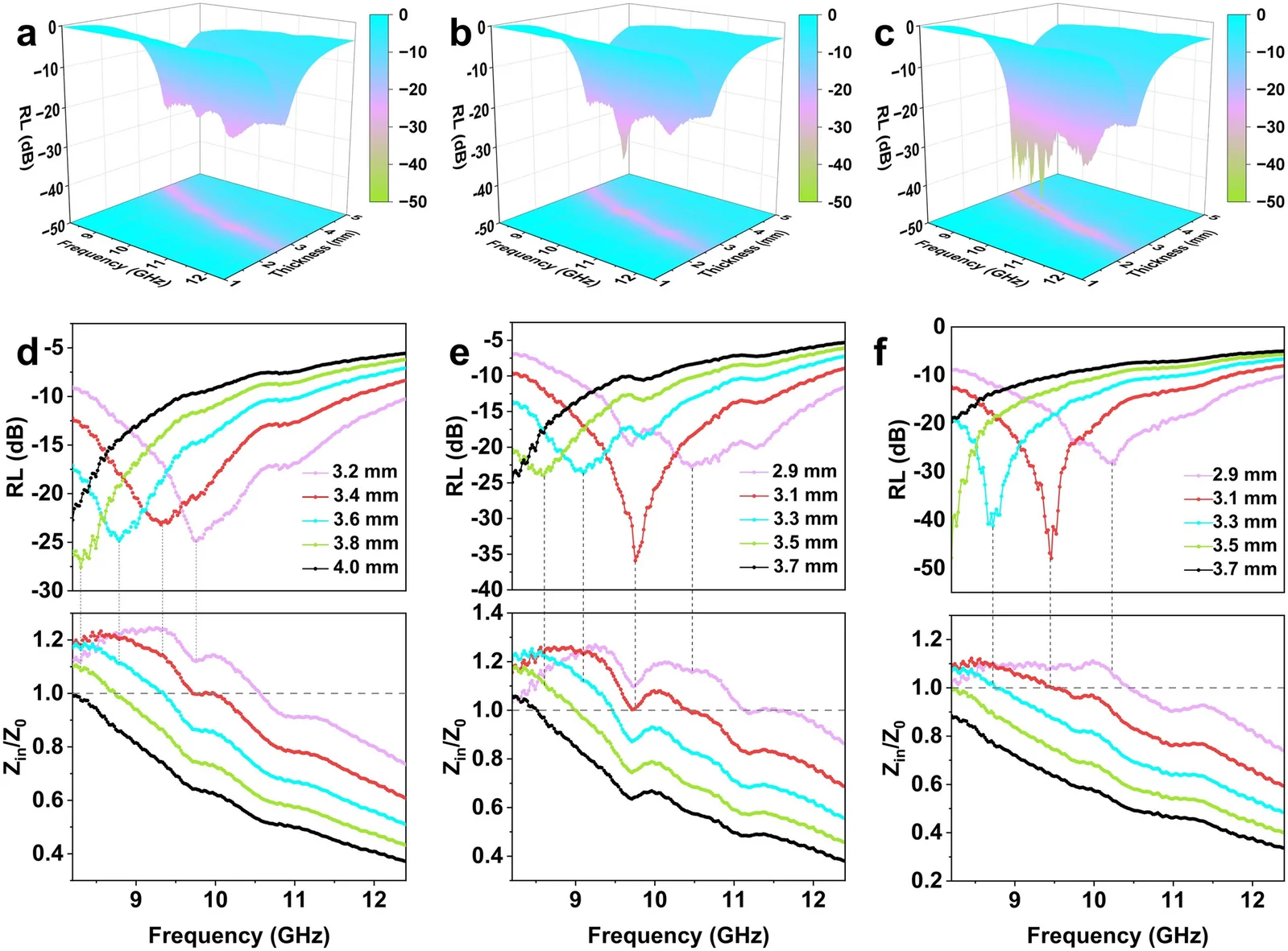
3D RL model of **a** blank gel, **b** MXene gel and **c** MXene/CDs gel. The RL and impedance matching of **d** blank gel, **e** MXene gel and **f** MXene/CDs gel

The electromagnetic wave absorption performance of a sample is typically evaluated by its reflection loss (RL), which is calculated from the complex permittivity and permeability parameters based on transmission line theory [46]. A RL value below − 10 dB indicates that 90% of incident electromagnetic energy is absorbed, corresponding to the effective absorption bandwidth (EAB), while a RL value below − 20 dB corresponds to 99% absorption efficiency [47]. As shown in Fig. 6a–c, the 3D RL plots clearly illustrate the absorption performance of the three gels. The blank gel exhibits a RL_min_ of − 27.55 dB at 3.8 mm thickness. In comparison, the MXene gel achieves an enhanced RL_min_ of − 35.88 dB at 9.75 GHz, with the optimal matching thickness reduced to 3.1 mm, demonstrating improved absorption efficiency together with reduced thickness. Notably, the MXene/CDs gel maintains the same matching thickness of 3.1 mm while achieving a significantly lower RL_min_ of − 47.9 dB at 9.46 GHz, along with a broad EAB of 3.5 GHz. Furthermore, a comparison of the reflection loss at the same thickness (Fig. S10) reveals that the MXene/CDs gel consistently outperforms the other samples, particularly at lower thicknesses. These results confirm that the incorporation of CDs into MXene effectively enhances microwave absorption capability through synergistic dielectric interactions. In addition to dielectric loss capability, efficient microwave absorption critically depends on impedance matching, which is quantitatively described by the normalized input impedance ratio (|*Z*_in_/*Z*_0_|). When |*Z*_in_/*Z*_0_| approaches 1, electromagnetic waves can enter the material efficiently rather than being reflected at the interface [48]. As shown in Fig. 6d–f, the blank gel exhibited a |*Z*_in_/*Z*_0_| value that slightly deviates from 1 at its RL_min_, indicating suboptimal impedance matching and partial reflection of incident waves. In contrast, the MXene/CDs gel exhibits a |*Z*_in_/*Z*_0_| value very close to 1 at its RL_min_, demonstrating excellent impedance matching performance. A comparison with recently reported microwave-absorbing materials, including hydrogels, aerogels, and polyurethanes, reveals that the MXene/CDs gel developed in this work exhibits superior absorption performance within the X-band (Table S1).

**Fig. 7:**
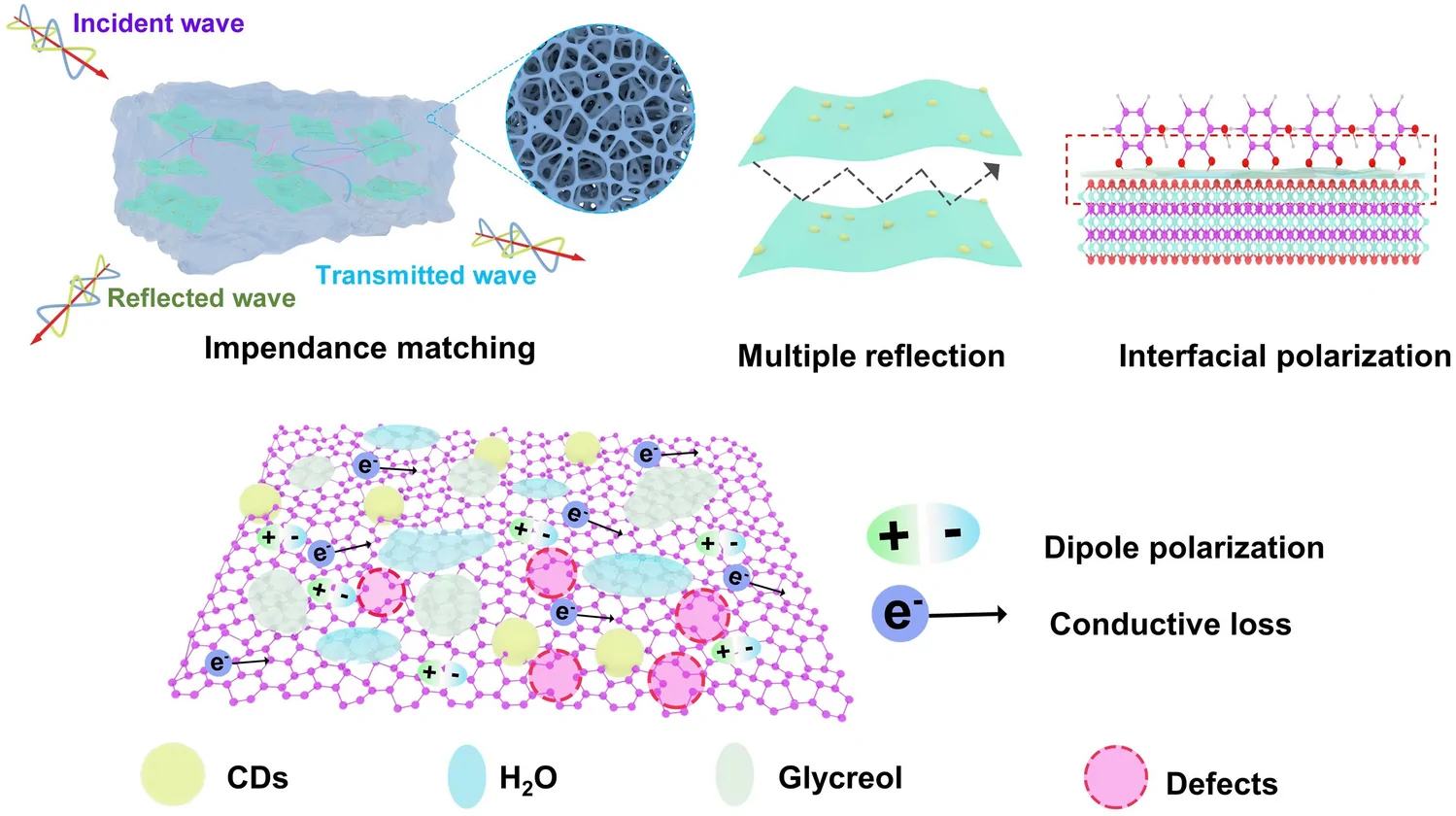
The schematic illustration for microwave absorbing mechanism of MXene/CDs gel

Figure 7 illustrates the proposed electromagnetic wave absorption mechanism of the MXene/CDs gel. The improved microwave absorption originates from the synergistic contributions of multiple mechanisms. The 3D porous network structure of the gel provides favorable impedance matching, allowing electromagnetic waves to enter into the interior of the material for subsequent attenuation and energy dissipation [49]. Within this interconnected structure, electromagnetic waves undergo multiple reflections and scattering among MXene nanosheets and polymer chains, effectively extending the propagation path and residence time of electromagnetic waves and thereby enhancing overall dielectric loss. The high electrical conductivity of the MXene/CDs composite contributes plays a crucial role in conductive loss. Meanwhile, abundant heterogeneous interfaces within the gel generate interfacial polarization due to the difference in dielectric properties between adjacent phases. Charge accumulation and relaxation at these interfaces lead to the formation of electric dipole and polarization loss. The incorporation of CDs, with their high specific surface area, further enhances the interfacial polarization by introducing additional active interfaces. Moreover, numerous dipoles are present in the system, including polar water and glycerol molecules, surface functional groups on the MXene and CDs, and dipoles associated with crystal defects within the conductive network. Under high-frequency electromagnetic fields, these dipoles undergo continuous reorientation and oscillation, converting electromagnetic energy into thermal energy through dipole polarization relaxation. Collectively, the synergistic effects of efficient impedance matching, multireflection effects, conductive loss, dipole polarization and interfacial polarization endow the MXene/CDs hydrogel with outstanding electromagnetic wave absorption performance.

## Conclusions

In this study, carbon dots (CDs) were synthesized from tannic acid (TA) via a directional ultrasound-assisted bottom-up approach and subsequently chemically anchored onto MXene nanosheets to fabricate MXene/CDs organic hydrogel absorbers. The incorporation of CDs increased the heterogeneous interfaces within the filler system, thereby enhancing interfacial polarization and dielectric loss. Meanwhile, the CDs acted as intercalation agent to expand the interlayer spacing of MXene and modulated its electrical conductivity. Together with an optimized gel solvent system, these effects contributed to improved impedance matching. Benefiting from the synergistic effect between dielectric loss and impedance matching, the MXene/CDs gel exhibited a RL_min_ of − 47.9 dB at 9.46 GHz. By adjusting the absorber thickness, the effective absorption bandwidth was extended to fully cover the entire X-band, demonstrating excellent microwave absorption performance. In addition, the organic hydrogel matrix endowed the absorber with favorable mechanical robustness. This work provides a viable strategy for constructing CDs/ MXene composites and offers insights into the rational design of flexible microwave absorbers with enhanced dielectric and mechanical properties.

## Supplementary Information

Below is the link to the electronic supplementary material.

Supplementary file1 (DOC 979 kb)
