# Supplementary material for: Carbon Dots Intercalated MXene for Flexible Organic Hydrogel Absorbers with Synergistically Enhanced Dielectric Loss
Source: Nanomicro Lett. 2026 Mar 25;18:302. doi: 10.1007/s40820-026-02135-6 (PMC13018520; doi:10.1007/s40820-026-02135-6)
Supplement: Supplementary file 1 — Supplementary file1 (DOC 979 kb) [file 40820_2026_2135_MOESM1_ESM.doc]

Supporting Information for

**Carbon Dots Intercalated MXene for Flexible Organic Hydrogel Absorbers with Synergistically Enhanced Dielectric Loss**

Bokai Lu^1^, Guangkai Jin^1^, Yuhong Cui^1^, Tianyi Zhang^1^, Shujuan Liu^1^, Qian Ye^1^, *, Xuqing Liu^1^, *, Feng Zhou^1, 2^

^1^ State Key Laboratory of Solidification Processing, Center of Advanced Lubrication and Seal Materials, School of Materials Science and Engineering, Northwestern Polytechnical University, Xi'an 710072, P. R. China

^2^ State Key Laboratory of Solid Lubrication, Lanzhou Institute of Chemical Physics, Chinese Academy of Sciences, Lanzhou 730000, P. R. China

^*^Corresponding authors. E-mail: [yeqian213@nwpu.edu.cn](mailto:yeqian213@nwpu.edu.cn) (Qian Ye); [xqliu@nwpu.edu.cn](mailto:xqliu@nwpu.edu.cn) (Xuqing Liu)

**S1 Experimental section**

**S1.1 Characterization**

The 2D layered structure and surface morphology of MXene, MXene/CDs, and the hydrogels were examined using scanning electron microscopy (SEM, ZEISS Sigma 300 and TESCAN MIRA). The morphology and microstructure of these samples were characterized by double spherical aberration-corrected transmission electron microscopy (Themis Z) and field-emission transmission electron microscopy (TEM, Talos F200X). The thickness of MXene nanosheets was measured using atomic force microscopy (AFM, Dimension Icon). Crystal structure information was obtained by X-ray diffraction (XRD, Rigaku SmartLab SE). Surface chemical compositions of these samples were analyzed by X-ray photoelectron spectroscopy (XPS, PHI 5000 VersaProbe III). The UV-Vis absorbance spectra of TA and CDs were measured using a UV-Vis spectrophotometer (LAMBDA 365). The internal interactions within the organic hydrogels were analyzed by Fourier transform infrared spectroscopy (FT-IR, Thermo Fisher Scientific Nicolet iS20). Changes in the surface charge of these samples were evaluated using a Malvern Zetasizer Nano ZS. The composition and degree of carbonization of MXene and MXene/CDs were investigated by Raman spectroscopy (WITec Alpha300R). The mechanical properties of the hydrogels were assessed at room temperature using a computer-controlled electronic universal testing machine (CMT2502). Electromagnetic parameters of the obtained composites were measured by the waveguide method using a vector network analyzer (Anritsu MS4644A). The electrical conductivity of the samples was measured at room temperature using an ST2643 ultra‑high‑resistance micro‑current tester equipped with an ST2643‑F01 standard annular three‑electrode probe.

**S1.2 Microwave absorption test**

According to the transmission line theory, attenuation constant can be expressed as [S1]:


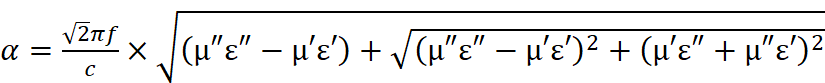
 (S1)

Where f represents the frequency of electromagnetic wave, c is the speed of light.

RL and impedance matching (Z) can be calculated through the following formulas [S2, S3]:


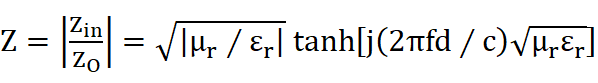
  (S2)


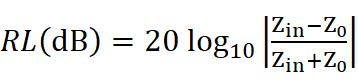
 (S3)

Where Z_in_ is the input impedance of absorber, Z_0_ is the impedance of free space,d is the thickness of absorber.

**S2 Supplementary Figures and Tables**


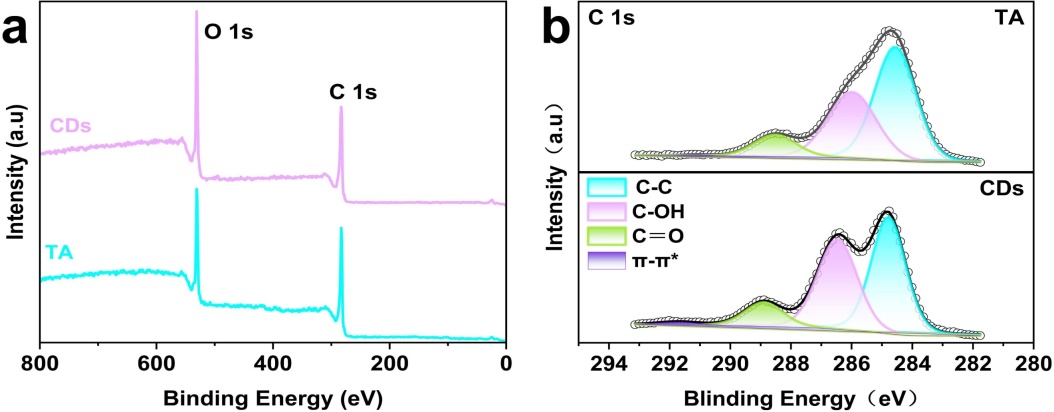


**Fig. S1** **a** XPS full spectra of TA and CDs. **b** High-resolution XPS spectra of O 1s of TA and CDs


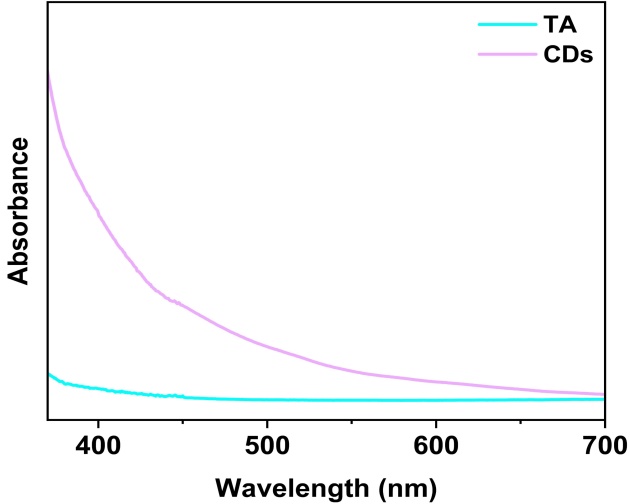


**Fig. S2** UV-Vis spectra of TA and CDs solution


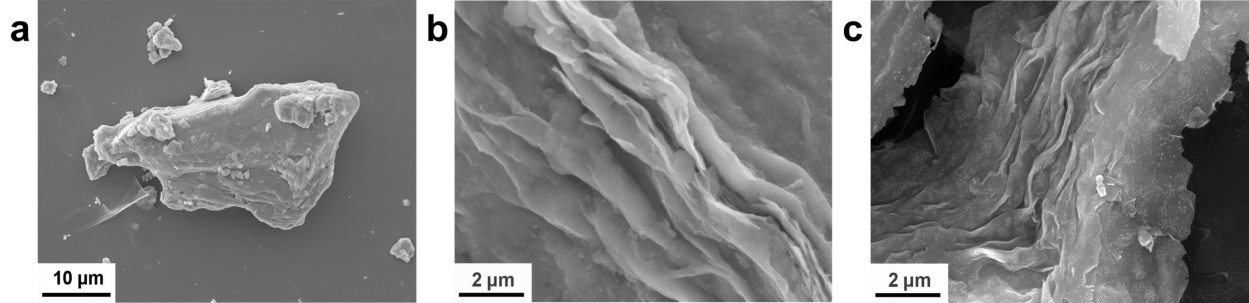


**Fig. S3** SEM images of **a** MAX phase, **b** MXene and **c** MXene/CDs


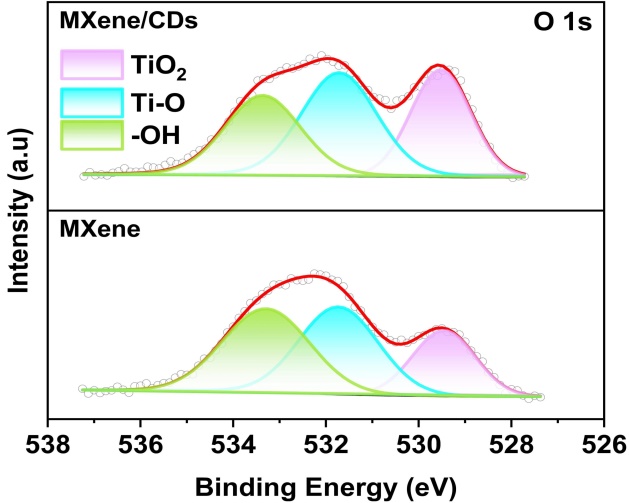


**Fig. S4** High-resolution XPS spectra of O 1s of MXene and MXene/CDs


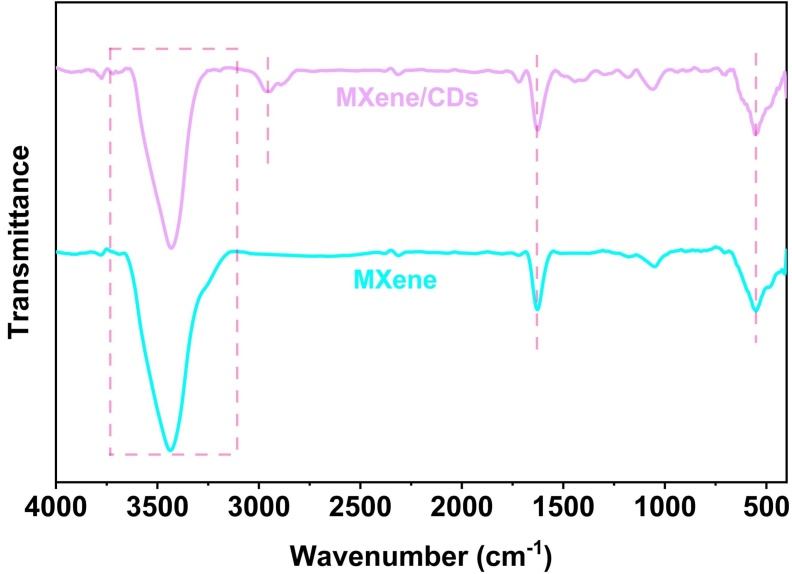


**Fig. S5** FTIR spectra of MXene and MXene/CDs


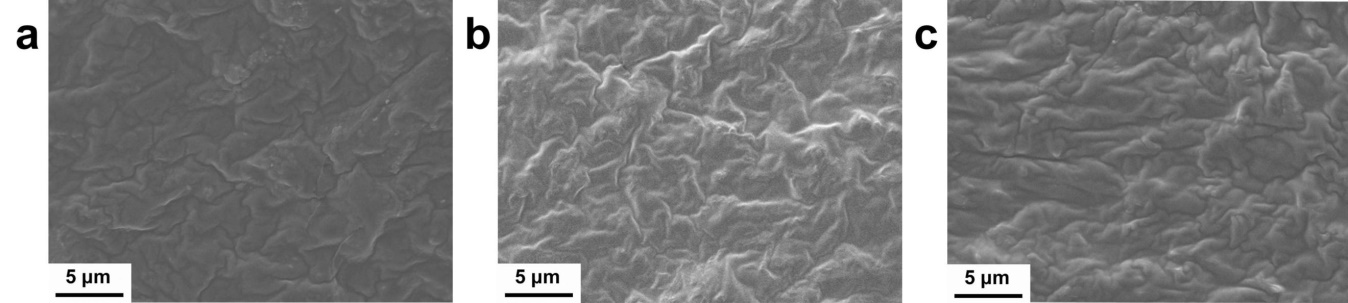


**Fig. S6** SEM images of **a** blank gel, **b** MXene gel and **c** MXene/CDs gel


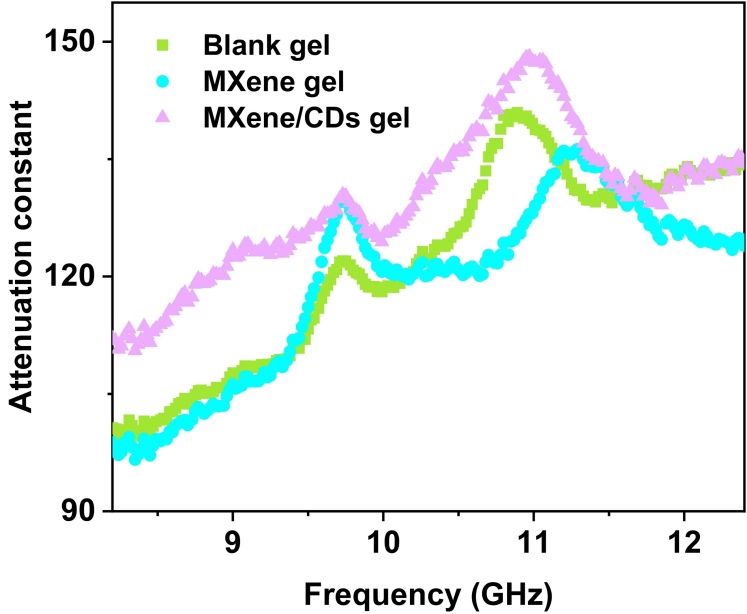


**Fig. S7** The attenuation constant of blank gel, MXene gel and MXene/CDs gel


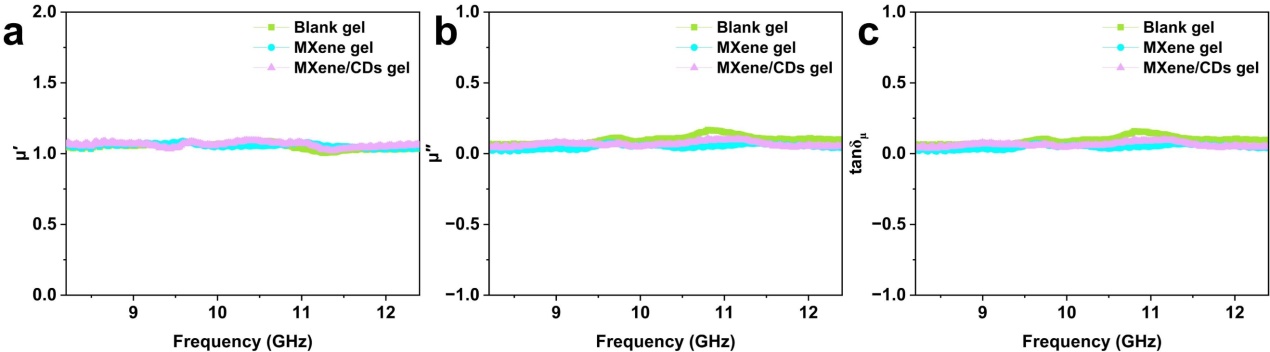


**Fig. S8** The **a** μ ', **b** μ'' and **c** tanδ_μ_ of blank gel, MXene gel and MXene/CDs gel


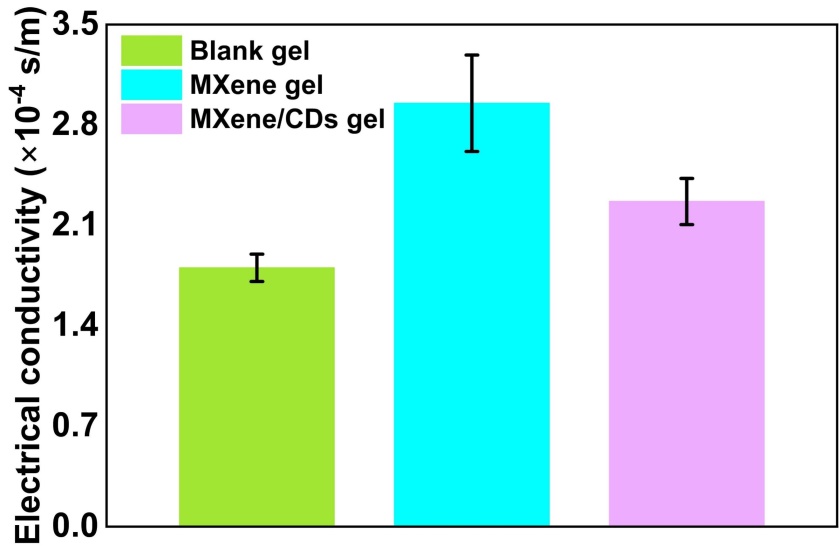


**Fig. S9** The electrical conductivity of blank gel, MXene gel and MXene/CDs gel


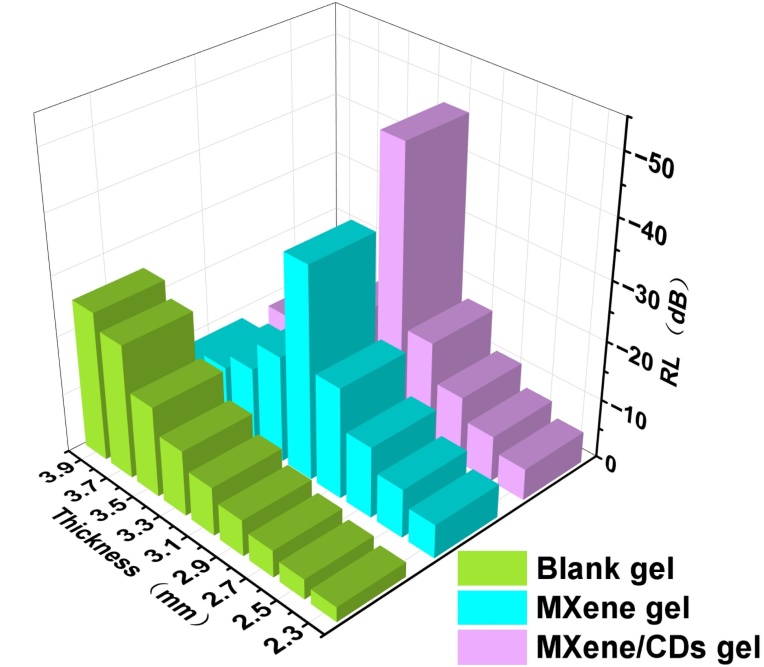


**Fig. S10** RL comparison of blank gel, MXene gel and MXene/CDs gel at the same thickness

**Table S1** Typical absorbing material and their microwave absorbing properties

| Materials | Thickness  (mm) | EAB  (GHz) | RL_min_ (dB) | References |
| --- | --- | --- | --- | --- |
| A_6_G_20_T_20_-2 hydrogel | 2.7 | 4.2 | -33.8 | [S4] |
| Magnetic cationic hydrogel | 2.5 | 5.2 | -45.9 | [S5] |
| PI/MXene-S aerogel | 2.5 | 5.4 | -33.5 | [S6] |
| ZIF hydrogel | 2.19 | 6.59 | -23.1 | [S7] |
| rGO-MCNTs-Fe_3_O_4_ hydrogel | 2.0 | 11.4 | -36 | [S8] |
| Acid-doped PCPU | 2.5 | 5.3 | -27.9 | [S9] |
| MXene/CDs hydrogel | 3.1 | 3.5 | -47.9 | This work |

**Supplementary References**

1. Y. Ma, Y. Jiang, C. Wang, B. Bao, G. Chen et al., Prune-like MoS_2_/CoS/GNs composites: a potential ultra-thin wave absorber. J. Alloys Compd. **971**, 172679 (2024). <https://doi.org/10.1016/j.jallcom.2023.172679>
2. M. Qin, L. Zhang, H. Wu, Dual-template hydrothermal synthesis of multi-channel porous NiCo_2_O_4_ hollow spheres as high-performance electromagnetic wave absorber. Appl. Surf. Sci. **515**, 146132 (2020). <https://doi.org/10.1016/j.apsusc.2020.146132>
3. X. Zhang, Z. Jia, F. Zhang, Z. Xia, J. Zou et al., MOF-derived NiFe2S4/Porous carbon composites as electromagnetic wave absorber. J. Colloid Interface Sci. **610**, 610–620 (2022). <https://doi.org/10.1016/j.jcis.2021.11.110>
4. Y. Zhang, L. Zhang, B. Zhou, Y. Gao, B. Zhang, Polarization-driven multifunctional organohydrogels with strain sensitivity toward electromagnetic wave absorption. Nano Res. **17**(6), 5688–5697 (2024). <https://doi.org/10.1007/s12274-024-6403-0>
5. H. Yuan, Y. Zhang, G. Lu, F. Chen, T. Xue et al., Transparent organogel based on photopolymerizable magnetic cationic monomer for electromagnetic wave absorbing. J. Ind. Eng. Chem. **109**, 538–546 (2022). <https://doi.org/10.1016/j.jiec.2022.02.039>
6. W. Zhang, E. Ding, W. Zhang, J. Li, C. Luo et al., Microstructure controllable polyimide/MXene composite aerogels for high-temperature thermal insulation and microwave absorption. J. Mater. Chem. C **11**(28), 9438–9448 (2023). <https://doi.org/10.1039/D3TC01210G>
7. H. Huang, G. Chen, Z. Li, S. Hui, L. Zhang et al., Constructing multi-polarization in metal–organic framework gels for electromagnetic wave absorption *via* ethanolamine hetero-coordination. Appl. Surf. Sci. **677**, 161052 (2024). <https://doi.org/10.1016/j.apsusc.2024.161052>
8. H. Zhang, M. Hong, P. Chen, A. Xie, Y. Shen, 3D and ternary rGO/MCNTs/Fe_3_O_4_ composite hydrogels: Synthesis, characterization and their electromagnetic wave absorption properties. J. Alloys Compd. **665**, 381–387 (2016). <https://doi.org/10.1016/j.jallcom.2016.01.091>
9. S. Yuan, J. Yang, Study on the mechanism, properties, and application asEMW-absorbing matrix materials of protonic acid-doped heat-resistant conductive polyurethanes. J. Appl. Polym. Sci. **143**, e57959 (2026). <https://doi.org/10.1002/app.57959>
